# Supplementary material for: Modeling glioblastoma heterogeneity as a dynamic network of cell states
Source: Mol Syst Biol. 2021 Sep 16;17(9):e10105. doi: 10.15252/msb.202010105 (PMC8444284; doi:10.15252/msb.202010105)
Supplement: Supplementary file 6 — Source Data for Figure 5 [file MSB-17-e10105-s004.zip › Figure5A_sourcedata/GSEA_3071/nosigdb_stateI.GseaPreranked.1622795145664/SUVA_MES1_PMID_31327527.html]

Details for gene set SUVA\_MES1\_PMID\_31327527[GSEA]

|  || Dataset | state13071 |
| Phenotype | NoPhenotypeAvailable |
| Upregulated in class | na\_pos |
| GeneSet | SUVA\_MES1\_PMID\_31327527 |
| Enrichment Score (ES) | 0.6871687 |
| Normalized Enrichment Score (NES) | 3.0553524 |
| Nominal p-value | 0.0 |
| FDR q-value | 0.0 |
| FWER p-Value | 0.0 |
Table: GSEA Results Summary

  

Fig 1: Enrichment plot: SUVA\_MES1\_PMID\_31327527      
 Profile of the Running ES Score & Positions of GeneSet Members on the Rank Ordered List

  

| PROBE | GENE SYMBOL | GENE\_TITLE | RANK IN GENE LIST | RANK METRIC SCORE | RUNNING ES | CORE ENRICHMENT || 1 | SPP1 |  |  | 0 | 2.808 | 0.1070 | Yes |
| 2 | MGP |  |  | 1 | 2.652 | 0.2080 | Yes |
| 3 | FN1 |  |  | 11 | 1.769 | 0.2728 | Yes |
| 4 | IGFBP7 |  |  | 45 | 1.387 | 0.3161 | Yes |
| 5 | TIMP1 |  |  | 47 | 1.381 | 0.3684 | Yes |
| 6 | APOE |  |  | 60 | 1.294 | 0.4143 | Yes |
| 7 | NNMT |  |  | 88 | 1.132 | 0.4497 | Yes |
| 8 | EMP1 |  |  | 122 | 1.012 | 0.4787 | Yes |
| 9 | CTSB |  |  | 129 | 0.964 | 0.5137 | Yes |
| 10 | NPC2 |  |  | 139 | 0.943 | 0.5471 | Yes |
| 11 | MGST1 |  |  | 150 | 0.915 | 0.5791 | Yes |
| 12 | EFEMP1 |  |  | 169 | 0.876 | 0.6073 | Yes |
| 13 | EMP3 |  |  | 260 | 0.726 | 0.6091 | Yes |
| 14 | GSN |  |  | 265 | 0.720 | 0.6353 | Yes |
| 15 | IFITM3 |  |  | 268 | 0.714 | 0.6620 | Yes |
| 16 | S100A11 |  |  | 275 | 0.707 | 0.6872 | Yes |
| 17 | LGALS3 |  |  | 390 | 0.600 | 0.6773 | No |
| 18 | NAMPT |  |  | 456 | 0.544 | 0.6794 | No |
| 19 | S100A10 |  |  | 673 | 0.422 | 0.6334 | No |
| 20 | GJA1 |  |  | 727 | 0.403 | 0.6335 | No |
| 21 | CLIC4 |  |  | 800 | 0.382 | 0.6274 | No |
| 22 | VIM |  |  | 1105 | 0.305 | 0.5517 | No |
| 23 | GFPT2 |  |  | 1229 | 0.283 | 0.5272 | No |
| 24 | ACTN1 |  |  | 1364 | 0.258 | 0.4985 | No |
| 25 | CD44 |  |  | 1430 | -0.251 | 0.4894 | No |
| 26 | A2M |  |  | 1808 | -0.295 | 0.3924 | No |
| 27 | MT2A |  |  | 2910 | -0.547 | 0.0970 | No |
| 28 | MT1E |  |  | 2982 | -0.591 | 0.0992 | No |
| 29 | SERPINE1 |  |  | 3050 | -0.634 | 0.1041 | No |
| 30 | S100A16 |  |  | 3155 | -0.736 | 0.1022 | No |
Table: GSEA details [plain text format]

  

Fig 2: SUVA\_MES1\_PMID\_31327527: Random ES distribution      
 Gene set null distribution of ES for **SUVA\_MES1\_PMID\_31327527**
